# Dairy by-Products Concentrated by Ultrafiltration Used as Ingredients in the Production of Reduced Fat Washed Curd Cheese

**DOI:** 10.3390/foods9081020

**Published:** 2020-07-30

**Authors:** Ana Raquel Borges, Arona Figueiroa Pires, Natalí Garcia Marnotes, David Gama Gomes, Marta Fernandes Henriques, Carlos Dias Pereira

**Affiliations:** 1 Polytechnic Institute of Coimbra, College of Agriculture, Bencanta, 3045-601 Coimbra, Portugal; raquelborges81@gmail.com (A.R.B.); arona@esac.pt (A.F.P.); natali@esac.pt (N.G.M.); david@esac.pt (D.G.G.); mhenriques@esac.pt (M.F.H.); 2 Research Centre for Natural Resources, Environment and Society (CERNAS), Bencanta, 3045-601 Coimbra, Portugal

**Keywords:** whey, buttermilk, second cheese whey, ultrafiltration, reduced-fat cheese

## Abstract

In the following study, three different dairy by-products, previously concentrated by ultrafiltration (UF), were used as ingredients in the production of reduced-fat (RF) washed curd cheeses in order to improve their characteristics. Conventional full-fat (FF) cheeses (45% fat, dry basis (db)) and RF cheeses (20–30% fat, db) were compared to RF cheeses produced with the incorporation of 5% concentrated whey (RF + CW), buttermilk (RF + CB) or sheep second cheese whey (RF + CS). Protein-to-fat ratios were lower than 1 in the FF cheeses, while RF cheeses ranged from 1.8 to 2.8. The tested by-products performed differently when added to the milk used for cheese production. The FF cheese showed a more pronounced yellow colour after 60 and 90 days of ripening, indicating that fat plays an important role regarding this parameter. As far as the texture parameters are concerned, after 60 days of ripening, RF cheeses with buttermilk presented similar results to FF cheeses for hardness (5.0–7.5 N) and chewiness (*ca.* 400). These were lower than the ones recorded for RF cheeses with added UF concentrated whey (RF + CW) and second cheese whey (RF + CS), which presented lower adhesiveness values. RF cheeses with 5% incorporation of buttermilk concentrated by UF presented the best results concerning both texture and sensory evaluation.

## 1. Introduction

The firm texture observed in reduced-fat (RF) and low-fat (LF) cheeses is one of the major problems resulting from fat reduction [1]. Cheese structure can be described as a continuous protein network interrupted by dispersed fat globules, which originate weak points in the protein network. In RF/LF cheeses the para-casein network becomes denser, originating the development of a firm and rubbery texture that does not break down during mastication [2,3].

Fat also plays an important role in the development of cheese flavour and appearance. The loss of flavour in RF/LF cheeses results from the lack of precursors from the fat, the lack of fat as a solvent for flavouring compounds, or the differences in the physical structure of RF/LF cheeses that inhibit certain enzymatic reactions which are essential for the formation of flavouring compounds [4]. Moreover, the observed differences between FF and RF Cheddar cheeses are not solely owed to differences in the cheese matrix and flavour release, but also to differences in ripening biochemistry, which lead to an imbalance of many flavour-contributing compounds [5].

Despite the significant advances in understanding both the biochemical and physicochemical characteristics of RF and LF cheeses and the introduction of technological developments, it is still necessary to evaluate solutions with the potential to improve the flavour, texture and sensory properties of such cheeses.

### Strategies to Improve the Characteristics of RF/LF Cheeses

The general approaches that have been used to improve the texture of RF or LF cheese involve decreasing the protein concentration, stimulating proteolysis, or creating a bigger filler phase to limit the density of the para-casein network [6]. These strategies can be divided into three categories: (i) manipulation of process parameters to enhance the moisture level; (ii) selection of specific starter cultures and use of adjunct cultures (i.e., non-starter lactic acid bacteria); and (iii) the use of stabilizers and fat mimetics to improve cheese texture [7].

The process parameters can be modified in order to increase water retention in the curd, which influences texture properties. This can be achieved by using lower coagulation temperatures; increasing the curd grain size; lowering curd scalding temperatures; or by increasing the surface area of the fat globules through milk homogenization [4,8].

The use of starter cultures that produce exopolysaccharide (EPS) can also improve the textural characteristics of LF cheeses by changing the microstructure and proteolysis [9] and has the potential to improve cheese flavour [10,11].

Fat replacers, alone or associated to the manipulation of process parameters, are the most promising alternative for improving the sensory properties of RF/LF cheeses. These ingredients are water-soluble compounds used to replace the functional characteristics of fat. They improve texture and cheese yield as well as the sensory and functional properties by binding water and providing a sense of lubricity and creaminess [12]. Polysaccharides and whey proteins (WP) are the most commonly used fat replacers.

Several reports evaluate the chemical and rheological properties of RF cheese with added polysaccharides as fat replacers. Guar and Arabic gums were tested on Iranian white cheese [13]. Waxy maize starch also increased the moisture content and water holding capacity, improving the overall quality of RF cheeses [14]. The partial replacement of milk fat for inulin increased meltability, cohesiveness and viscosity, while decreasing hardness and adhesiveness of acid casein processed cheeses [15]. Carrageenans also improved the textural and rheological properties of LF cheeses by increasing moisture in non-fat solids (MNFS) [16]. Cheeses containing agave fructans were compared to FF and RF samples without fructans and demonstrated the texturing role of the carbohydrates [17]. Konjac glucomanan was also indicated as a potential fat replacer to be used in Mozzarella [18]. The addition of alginate also improved the textural, microstructural and colour properties of LF Cheddar cheeses [19]. Recently waxy rice starch, sodium carboxymethyl cellulose (CMC) and glutamine transaminase were also tested as texturizers and crosslinking agents in Mozzarella [20].

Native WP can be aggregated to obtain colloidal microparticulated whey protein (MWP). MWP is formed by mixing native whey proteins and protein aggregates. These particles can be manufactured in diameters ranging from 0.1 to 100 µm [21,22]. Their ability to enhance creaminess is based on a “ball bearing mechanism” originally reported by Cheftel and Dumay [23] and recently confirmed [24].

Several aspects have to be pondered when using WP as ingredients for cheese production, such as: (i) the importance of WP denaturation, which allows their contribution to the protein matrix of the cheese; (ii) the increased water-holding capacity of cheese curds; (iii) the lower acidification of the cheeses as a result of the higher buffering capacity of WP; (iv) the occurrence of differences in flavour of the modified products, which tend to be more pronounced during ripening [25].

When using WP as a fat replacer in cheese it is recommended a low ratio of native/denatured WP to ensure its function as an inert filler. It has also been ascertained that particle size should be within the range of 1 to 10 µm in order to avoid disturbance of the para-casein network [26]. However, other authors advocate that higher particle sizes (20–100 µm) do not impart negative effects to cheese properties, namely taste, flavour or consistency [27,28].

Whey proteins and MWP are commercially available as powders. However, small and medium-scale cheese plants can concentrate whey by ultrafiltration and, after proper treatments, they can use the liquid whey concentrates in the cheese production. The same methodology can be applied to other dairy by-products, allowing for their in-plant valorisation. Hence, in the present work, we selected three liquid by-products of the dairy industry, namely: buttermilk, whey and sheep second cheese whey (the by-product resulting from the production of whey cheese), all previously concentrated by ultrafiltration (UF), to be tested as ingredients in the production of experimental RF/LF cheeses.

## 2. Materials and Methods

The three reduced-fat, cow’s milk cheeses produced with different dairy by-products as ingredients were compared with conventional full-fat cheese (FF) (>45% fat db) and conventional reduced-fat (or half-fat) cheese (RF) (25–45% fat db) (without any addition). The tested fat replacers were, buttermilk (CB), whey (CW) and sheep’s second cheese whey (CS).

Second cheese whey, known as *Sorelho*, is the by-product resulting from the manufacture of *Requeijão*, the Portuguese whey cheese, which is produced by thermal aggregation of whey proteins (at ca. 90 °C for 20 min). This product still contains approximately 50% of the original dry matter of the original whey. Lactose and minerals largely contribute to its dry mass, but residual fat and non-thermally precipitated nitrogen components are still present.

All the by-products were previously concentrated by ultrafiltration (UF) with a volumetric concentration factor of ca. 15 (VCF = VFeed/VRetentate). The five cheese products were coded as: (i) conventional full-fat cheese (FF); (ii) reduced-fat cheese (RF); (iii) reduced-fat cheese with buttermilk (RF + CB); (iv) reduced-fat cheese with whey (RF + CW) and; (v) reduced-fat cheese with sheep second cheese whey (RF + CS).

UF concentrates were obtained by following the procedure described by Henriques and co-workers [25] although with small adjustments, namely the ultrafiltration process temperature (40–45 °C) and the smaller membrane cut-off (10 kDa). In the case of CW, after concentration, the retentate was submitted to thermal denaturation (90 °C for 20 min) prior to homogenization at 10 MPa, in a homogenizer Rannie™ model Blue Top (APV, Albertslund, Denmark) and kept frozen (−25 °C) until the moment of use. Buttermilk and *Sorelho* were concentrated by UF using the same conditions and pasteurized at 75 °C for 5 min and kept frozen at −25 °C prior to their incorporation into the milk.

The bovine milk (Quinta da Cioga, Portugal) was delivered to the dairy pilot plant at *Escola Superior Agrária de Coimbra* (ESAC). Part of the milk was skimmed in a Westfalia™ type ADB centrifuge (Westfalia Separator, Germany) and standardised to 3.5% (*v*/*v*) of fat (for the production of FF cheese) and to 1.5% (*v/v*) of fat (for the production of RF cheeses). At this stage, according to the batches to be produced, the fat replacers (5% *v/v*) were added. Each batch was made up of 40 L.

Milk pasteurization took place at 74 ± 1 °C for 30 s in Pasilac Therm™ (PHE Nordic, Denmark) plate and frame heat exchanger. After temperature stabilization of the mixtures at 29.5 ± 0.5 °C, 0.2 mL^−1^ CaCl_2_ solution (51% *w/v*) (supplied by Tecnilac, Portugal), starter culture (Mesófilo Plus Starter, Enzilab, Portugal) (10 mgL^−1^, containing *Lactococcus lactis* subsp. *lactis, Lactococcus lactis* subsp. *cremoris* and *Streptococcus thermophilus*), KNO_3_ (25 mgL^−1^) and 20 mgL^−1^ animal rennet (>92 g/100 g chimosin, supplied by Tecnilac, Portugal) were added to the milk formulations and mixed thoroughly. The coagulation of the mixtures was performed for approximately 45 min at 30 ± 1 °C. When coagulation was completed, grids were used to cut the curd into small pieces (2 cm^3^), in order to promote whey drainage. After draining half of the whey, the same amount of salted hot water (1% *w/v* salt, 30 °C) was added to the curd and the mixture was thoroughly agitated prior to the final whey drainage. The recovered curd was then placed into plastic moulds before being pressed and stored in a refrigerated chamber at 8–9 °C, for 24 h. After this period, cheeses, weighing approximately 250 g (ca. 4.5 cm height and 8.0 cm diameter) were immersed in a brine solution (18–20 °Baumé) for 1.5 h and finally transferred to the ripening chamber (10 ± 2 °C) being kept there for 90 days.

The chemical composition of cheeses, colour, texture, pH and titratable acidity were assessed on the 1st, 30th, 60th and 90th days of ripening. Each physicochemical parameter was evaluated in triplicate.

Cheese moisture was determined by drying the cheese sample in an oven at 105 °C for 24 h according to AOAC method 248.12 Dried samples were tested for ash content in a muffle furnace at 550 °C for 4 h (AOAC 935.42) [29].

The fat content was determined using the Van Gulik method (ISO 3433, 2008) [30]. Cheese protein was determined by multiplying the total nitrogen content of the samples, obtained using the Kjeldahl procedure (AOAC 920.132) [29], by a factor of 6.38.

The pH was measured directly from the cheeses, using a pH meter (PHM61 Laboratory pH Meter, Denmark) equipped with a probe for reading solids and the titratable acidity was expressed as g of lactic acid/100 g cheese (AOAC 920.124) [29].

According to the physicochemical composition of each cheese sample, moisture in non-fat solids (MNFS) and fat in dry matter (FDM) were calculated.

Colour was expressed by the individual three coordinates of CIE L*(lightness), a*(red-green axis) b*(blue-yellow axis) system using a Chroma Minolta CR-200B colorimeter (Japan). For each cheese type, three readings for colour were performed on the rind and on the paste of two cheeses (*n* = 6).

A Stable Micro Systems Texture analyzer, model TA.XT Express Enhanced (Stable Micro Systems, Surrey, UK), was used to perform textural analysis and the results were calculated by the Specific Expression PC Software. A texture profile analysis was run with a penetration distance of 15 mm at 1 mm/s test speed, using an acrylic cylindrical probe with a diameter of 12.5 mm and height of 38.1 mm. The following parameters were quantified: hardness (N) (the peak force measured during the first compression cycle), adhesiveness (g.s) (the negative force area for the first bite, representing the necessary work to pull the compression plunger away from the sample), cohesiveness (the ratio between the positive area during the second compression and the area during the first compression) and chewiness (the product of gumminess and springiness) were quantified. Three penetrations were performed on the surface (without rind) of two cheese samples (*n* = 6).

Ninety non-trained members of staff and students performed sensory analysis, at the 30th, 60th and 90th days of ripening. Each sensory evaluation test involved 30 members which, individually, expressed their consent to participate in the tests. The tests involved evaluation of the cheese samples according to the following parameters: external and sliced aspect, aroma, taste and texture. Overall impression was also evaluated through a ranking test. Each cheese category was coded and the tasters were asked to evaluate both the visual and gustatory aspects using a 1–9 scale (1 = dislike extremely; 2 = dislike very much; 3 = dislike moderately; 4 = dislike slightly; 5 = neither like nor dislike; 6 = like slightly; 7 = like moderately; 8 = like very much; 9 = like extremely).

One-way ANOVA tests, included in StatSoft Statistica 8.0 (Statsoft Iberica, Lisbon, Portugal), were performed to compare the means of the physicochemical properties of the cheeses and the attributes used for sensory evaluation. The Tukey HSD post-hoc test, with a 95% confidence level was applied to assess differences between treatments.

## 3. Results and Discussion

Table 1 presents the composition of the different ingredients added as fat replacers to the 1.5% (*v/v*) fat milk batches. Significant differences were observed in the protein, fat and ash contents of those products. Concentrated buttermilk presented the lowest level of protein, while CS presented the highest value. The lowest fat content was observed in CS. CB and CW presented similar dry matter values, while CS showed a significantly lower content.

The differences in the composition of the ingredients are reflected in the dry matter and fat content of the mixtures used for cheese production (Table 2). The mixtures containing CW and CS presented the lowest levels of fat, while the mixture used for the production of FF cheese presented a higher level of fat and lower levels of protein, lactose and minerals. The protein, lactose and ash contents did not show significant differences between the mixtures used for the production of RF cheeses.

**Figure 1:**
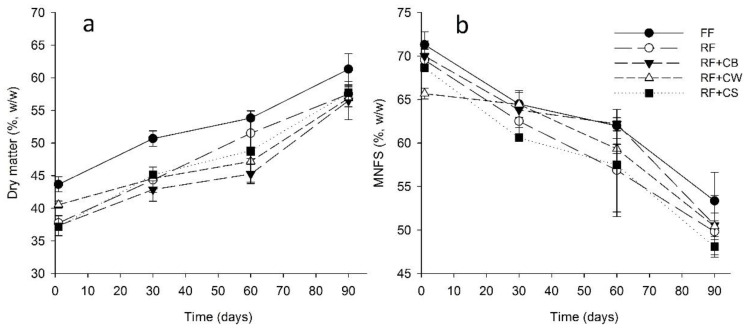
Dry matter (**a**) and moisture in non-fat solids (MNFS) (**b**) of tested cheeses over ripening. (FF): full-fat; (RF): reduced-fat; (RF + CB): reduced-fat with concentrated buttermilk; (RF + CW): reduced-fat with concentrated whey; (RF + CS): reduced-fat with concentrated second cheese whey.

As it can be observed in Figure 1a, in all stages of ripening, the FF cheese presented a significantly higher (*p* < 0.05) level of solids when compared to all other samples. Both the type of cheese and ripening time had significant effects on the dry matter content. Concerning moisture in non-fat solids (MNFS) FF cheeses also presented higher values (Figure 1b). Exceptions were the values of MNFS of RF, RF + CB and RF + CW at the 30th day of ripening. At the 60th day of ripening, RF + CB also presented values similar to FF.

According to the Portuguese standard, NP-1598 [31], at the 30th day of ripening all cheeses could be considered as semi-soft (61–69% MNFS). At the 60th day, only FF and RF + CB cheeses maintained this classification, being all others classified as semi-hard (54–63% MNFS). At the end of the ripening period (90th days), all the cheeses were classified as hard (49–56% MNFS).

**Figure 2:**
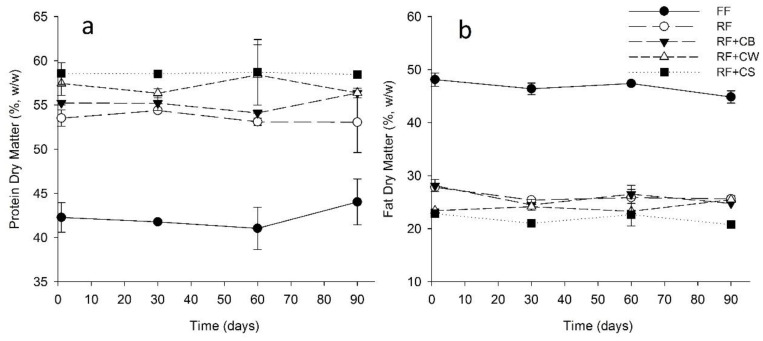
Protein in dry matter (**a**) and fat in dry matter (FDM) (**b**) of tested cheeses over ripening. (FF): full-fat; (RF): reduced-fat; (RF + CB): reduced-fat with concentrated buttermilk; (RF + CW): reduced-fat with concentrated whey; (RF + CS): reduced-fat with concentrated second cheese whey.

The protein content was significantly lower in the FF cheeses (Figure 2a). In all other cheese samples, protein represented more than 50% of the solids, having the highest values been observed in RF + CW and RF + CS. Concerning fat content (Figure 2b), the FF cheese presented more than 45% fat on dry basis (being classified as a full-fat cheese according to NP-1598), whereas all other cheeses presented values ranging from 20 to 30%. In the cases of RF and of RF + CB the cheeses could be classified as half-fat (25–45% dry basis) in all stages of ripening. Cheeses with values of fat in the range 10–25% are classified as low-fat. This is the case of RF + CS in all stages of ripening and of RF + CW until the 60th day of ripening.

**Figure 3:**
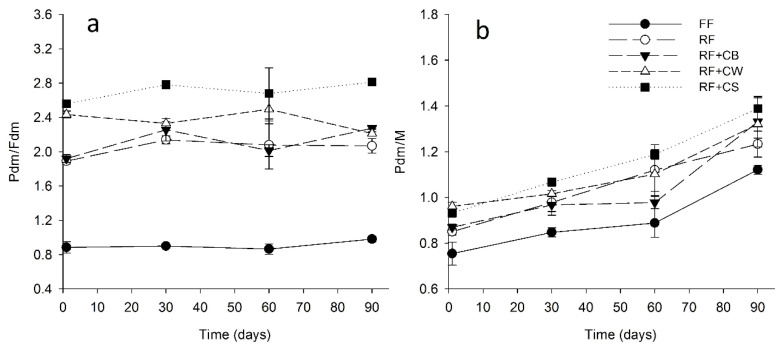
Ratio protein in dry matter/fat in dry matter (Pdm/Fdm) (**a**) and protein in dry matter/moisture (Pdm/M) (**b**) of tested cheeses over ripening. (FF): full-fat; (RF): reduced-fat; (RF + CB): reduced-fat with concentrated buttermilk; (RF + CW): reduced-fat with concentrated whey; (RF + CS): reduced-fat with concentrated second cheese whey.

The ratio protein in dry matter/fat in dry matter (Pdm/Fdm) (Figure 3a) showed clear differences between FF and RF cheese samples. This value was lower than 1 in the FF cheese, whereas in the case of the remaining samples it was in the range of 1.8–2.8, this being the highest value observed in RF + CS. Although CS presented significantly higher protein content when compared to CB or CW (Table 1), the mixtures used for cheese production did not show significant differences regarding this parameter. Hence, the protein content of CS cannot justify, on its own, the higher Pdm/Fdm ratio observed in RF + CS. Second cheese whey normally presents a high proportion of whey protein aggregates resulting from the drastic heat treatment (*ca.* 90 °C 10 min) to which whey is submitted during the production of whey cheeses. The better retention of such aggregates in the cheese curd, as compared to native proteins, may explain the higher protein content of RF + CS and, to some extent, of RF + CW (in which protein was also denatured). With regard to the ratio protein in dry matter/moisture (Pdm/M), the maximum value attained by the FF cheeses was around 1.1, at the end of the ripening period, whereas in the case of the reduced fat cheeses was in the range of 1.2–1.4 at the end of ripening (Figure 3b). Higher values of Pdm/Fdm and of Pdm/M are expected to promote a firmer texture, often associated to the lower sensory scores obtained by RF cheeses. All the RF cheeses presented ratios of Pdm/M higher than 1.0 after the 30th of ripening with the exception of RF + CB cheeses that maintained Pdm/M values lower than 1.0 until the 60th day of ripening. This fact had positive repercussions on the textural and sensory properties of these cheeses, comparing well with FF cheese.

**Figure 4:**
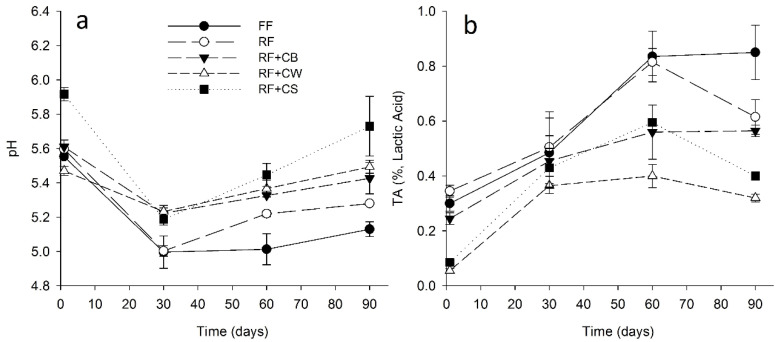
(**a**) pH and (**b**) titratable acidity (TA) of tested cheeses over ripening. (FF): full-fat; (RF): reduced-fat; (RF + CB): reduced-fat with concentrated buttermilk; (RF + CW): reduced-fat with concentrated whey; (RF + CS): reduced-fat with concentrated second cheese whey.

On the first day of ripening, the pH values of the cheeses were in the order of 5.4–5.9, being significantly lower (*p* < 0.05) in the case of RF + CW and significantly higher in the case of RF + CS (ca. 5.9) (Figure 4a). After the 30th day of ripening, the values decreased to 5.0 in the case of FF and RF, being significantly higher in the cases of cheeses with added fat replacers (ca. 5.2). After this moment, the pH increased steadily until the end of ripening, the increase was significantly (*p* < 0.05) more pronounced in the case of cheeses with added fat replacers. The RF + CS cheeses showed higher pH values at the 60th day of ripening, the results were significantly higher on the 90th day. The titratable acidity (TA) showed an opposite tendency (Figure 4b). In this case, the highest values were observed for FF and RF cheeses at the 60th and 90th days of ripening. Overall, the reduced-fat cheeses containing fat replacers presented lower TA values, these being the lowest values observed for RF + CW in all stages of ripening.

**Figure 5:**
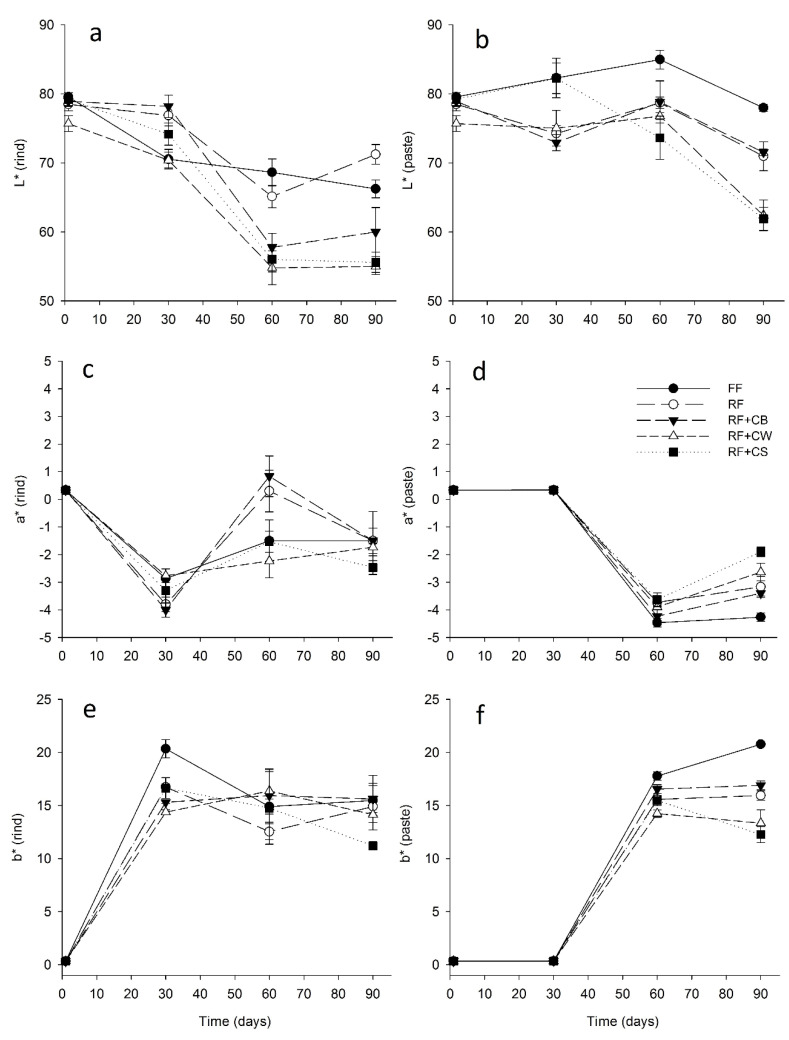
Colour parameters of the rind (**a**,**c**,**e**) and of the paste (**b**,**d**,**f**) of tested cheeses over ripening. (FF): full-fat; (RF): reduced-fat; (RF + CB): reduced-fat with concentrated buttermilk; (RF + CW): reduced-fat with concentrated whey; (RF + CS): reduced-fat with concentrated second cheese whey.

Concerning the colour parameters of the cheeses (Figure 5), the luminosity (L*) of the rind of RF + CW cheeses was significantly lower at the first day of ripening. At the 30th day, the L* value of the rind was significantly higher in RF and RF + CB when compared to all other samples. After the 60th day of ripening all the reduced-fat cheeses showed significantly lower L* values when compared to FF, having the cheeses with added fat replacers a clearly darker tone when compared to FF an RF. The luminosity of the paste showed a tendency to increase between the 30th and the 60th day, except in the case of RF + CS. At the end of the ripening period all cheeses showed significantly lower L* values when compared to the initial values, as a result of the progressive darkening of the paste. At the 90th day, all the reduced-fat cheeses showed significantly lower L* values of the paste, being the paste of RF + CW and RF + CS significantly darker than the ones of RF and RF + CB. Thus, it appears that both those fat replacers significantly impaired the colour of the cheeses paste. Concerning the a* parameter of the rind, the initial values were very similar, in short the initial white colour of the products shifted towards green (*ca.* −3.5) after 30 days. Then, these values increased until the 60th day, having the increase been more pronounced in RF and RF + CB. By the end of ripening the a* values of the rind decreased again in all reduced-fat cheeses, with the exception of RF + CW. At the end of ripening RF + CS showed a significantly lower a* value. The a* values of the paste were very similar until the 30th day, then decreased at the 60th day and finally increased slightly at the end of the ripening period. This increase was more pronounced in RF + CW and RF + CS. The FF cheese presented significantly lower a* values at the 90th day of ripening. From the 1st to the 30th days of ripening, the b* value of the rind shifted from 0 to around 20 in the case of the FF cheese, while in the cheeses with fat replacers, at the 30th day, the b* values were in the order of 14–16. This evolution indicates the shift from white to yellow. Then, the b* values were maintained, or slightly reduced, until the end of ripening. The same pattern was observed with the b* paste’s value. However, the change was only significant from the 60th day onwards. The paste of the FF cheese presented a more pronounced (*p* < 0.05) yellow colour at the 60th and 90th days of ripening, indicating that fat has a significant impact on this parameter. RF cheeses presented significantly lower values of b* after the 60th day of ripening. RF + CW and RF + CS presented the lowest values, while RF and RF + CB presented intermediate values.

**Figure 6:**
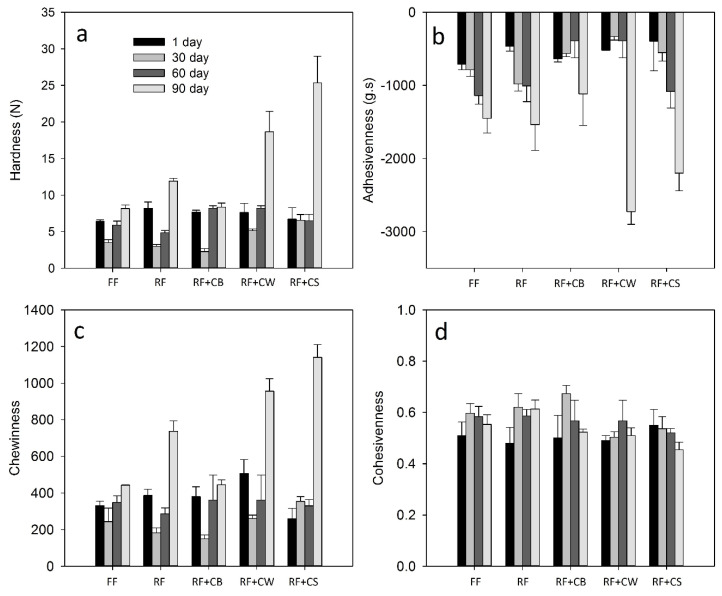
Texture parameters of the tested cheeses over ripening. (**a**) Hardness; (**b**) Adhesivenness; (**c**) Chewinness; (**d**) Cohesivenness. (FF): full-fat; (RF): reduced-fat; (RF + CB): reduced-fat with concentrated buttermilk; (RF + CW): reduced-fat with concentrated whey; (RF + CS): reduced-fat with concentrated second cheese whey.

In regards to the texture (Figure 6), hardness values showed significant differences (*p* < 0.05) at the 30th day of ripening, having the RF + CW and RF + CS presented slightly higher values. From the 60th to the 90th day of ripening, a significant increase of the hardness values was observed in the cases of RF, RF + CW and RF + CS, being less pronounced in RF. FF cheeses and RF + CB maintained values of hardness in the order of 5.0–7.5 N during the entire ripening. In the case of adhesiveness, RF + CW and RF + CS also presented significantly lower values by the end of the ripening period, whereas RF + CB presented the highest values, although not significantly different from FF and RF. In all cases, this parameter significantly decreased between the 60th and the 90th day of ripening. At the end of ripening, chewiness values were significantly higher in RF, RF + CW and RF + CS being the values of RF + CB similar to those of FF. It is evident that, after the 60th day of ripening, with the exception of RF + CB, all the reduced-fat cheeses presented clear differences in these texture parameters when compared to FF cheeses. Thus, it can be considered that the use of liquid buttermilk was the best option for the replacement of fat, since RF + CB cheeses are very similar to FF cheeses. Other authors reported that fat reduction increased the hardness of *Minas* fresh cheeses, promoting a denser microstructure and less proteolysis [14]. In a previous work we reported values in the order of 3.6 N for the hardness of RF cheeses with addition of 10 (*v/v*) liquid whey protein concentrates (LWPC) plus 0.25–0.5% (*m*/*v*) Simplesse™, while the conventional RF cheeses showed values in the order of 8 N. The cheeses with addition of LWPC also presented significantly higher levels of MNFS, when compared to conventional RF cheeses [25]. As reported by other authors, cheese fracturability and hardness increase with decreasing fat, while elasticity and adhesiveness decrease. Cheese lightness and red and yellow indexes also decrease with decreasing fat content, as it occurred with our samples [32].

The sensory evaluation results are depicted in Table 3 and Table 4. At the 30th day of ripening no differences between cheeses could be detected with regards to their appearance. RF + CW and RF + CS cheeses obtained significantly lower scores for texture and taste. This fact is also demonstrated by the lower ranking obtained by both samples (Table 4). At the 60th day of ripening the defects in texture and taste of RF + CW were not evident, while RF + CS showed significantly lower scores for these parameters. However, at the end of the ripening period RF + CW presented significantly lower scores for appearance, texture and taste. The FF cheese presented the highest scores for aroma and taste, at the 60th day of ripening, and for appearance and taste, at the end of the ripening period. RF + CB presents similar results to the FF cheeses in all stages of ripening. It should be highlighted that, at the 30th day of ripening, RF + CB cheeses obtained the highest scores, although not significantly different from the ones obtained by FF cheeses. The main defect reported by panelists was related to the hardness of RF + CW and RF + CS at the 90th day.

At the 60th day of ripening the ratio Pdm/M of RF + CB showed values similar to those of FF cheeses, while in all other cases this ratio presented significantly higher values, which adversely affected texture. It is reported by Skeie et al., 2013 that microparticulated whey protein and buttermilk added to the cheese milk improved the texture of RF cheeses, whereas the flavour could be improved by selected *Lactobacillus* spp. isolated from good-quality cheeses [33]. The results obtained by these authors showed that it was possible to produce a 10% fat Dutch cheese with an improved texture compared with the regular cheese without any additional ingredients. MWP also improved yield and the textural properties of RF Cheddar cheese due to the water-binding ability of denatured whey protein and by decreasing firmness [34]. Hence, similar results could be expected with the addition of concentrated cheese whey (CW). However, this was not observed, particularly after the 60th day of ripening. Perreault and co-workers assessed the effect of denatured whey protein concentrate (DWPC) and its fractions on cheese yield composition, and rheological properties of cheeses. For cheeses with the same moisture content, the use of DWPC had no direct effect on rheological parameters. The protein aggregates were primarily responsible for the increase in cheese yield while moisture content explained, to a large extent, the variation in cheese rheological properties [35]. Other authors evaluated the fat mimicking mechanism of MWP in milk-based systems using rheological and tribological techniques, and reported that friction levels attained with MPW proteins and dairy fat at typical speeds involved in oral processing were comparable, demonstrating therefore the capability of MWP dispersions to imitate dairy fat in milk-based systems from a lubrication point of view [36].

Regarding the use of buttermilk powder in LF Cheddar cheese, it is reported that cheese made with BM addition had a homogeneous protein network with small voids and a smoother and less coarse structure when compared to LF cheeses without buttermilk addition [37]. The addition of liquid BM to cheese milk was also tested. As the percentage of BM increased, the total solids, fat, protein, fat in dry matter and ash of cheese milk decreased significantly, leading to a softer and moister curd. However, samples prepared with more than 25% BM were not acceptable with respect to the taste panel [38]. The effects of BM powder addition post-curd formation, or liquid BM addition to cheese milk on the characteristics of Cheddar-style cheese were evaluated in parallel. Addition of 10% BM powder resulted in higher phospholipid content, moisture, pH and salt levels, and lower fat in dry matter. BM addition also originated a more porous cheese microstructure with higher fat globule coalescence and increased free fat, while increasing moisture and decreasing protein, fat and pH levels [39]. It is also reported that liquid BM addition to cheese milk resulted in a softer cheese compared to other cheeses, while BM powder addition had no influence on cheese firmness compared to the control cheese. However, significant differences in sensory profiles associated with off-flavour were also observed with the addition of liquid BM to cheese milk. Addition of 10% BM powder to cheese curds resulted in cheese comparable to the control Cheddar with similar structural and sensory characteristics, although with differences in overall cheese flavour [40]. In the case of our products no adverse effects resulted from the addition of BM to cheese milk and this fact may be attributed to the lower amount added.

## 4. Conclusions

The reduction of fat in cheeses often affects negatively their sensory properties. Therefore, several approaches are normally used to minimize those negative effects. In the present study, UF concentrated liquid buttermilk, whey protein concentrate, and sheep’s second cheese whey were used for this purpose. From the results obtained, it is evident that UF concentrated liquid buttermilk significantly improved the properties of RF/LF cheeses, which showed good overall sensory evaluation and compared well to FF cheeses. The use of UF concentrated dairy by-products can allow for their direct valorisation in dairy plants and represents a significant contribution to the circular economy. It is recommended that further work should compare the fat replacing properties of such products, both in the liquid and dry form. Optimization of mixtures of such by-products should also deserve further research.

## Figures and Tables

**Table 1:** Proximal composition of the different ingredients used for cheese production (% *w/v*).

Ingredients	Dry Matter	Protein	Fat	Ash
CB	13.78 ^a^ ± 0.42	3.65 ^a^ ± 0.07	1.41 ^a^ ± 0.01	0.75 ^a^ ± 0.09
CW	13.19 ^a^ ± 0.68	5.49 ^b^ ± 0.04	2.81 ^b^ ± 0.01	0.92 ^b^ ± 0.03
CS	10.35 ^b^ ± 0.14	6.56 ^c^ ± 0.05	0.41 ^c^ ± 0.01	0.54 ^c^ ± 0.02

(CB): UF concentrated buttermilk; (CW): UF concentrated whey; (CS): UF concentrated sheep’s second cheese whey. Means within the same column with different superscripts are significantly different (*p* < 0.05).

**Table 2:** Composition of the different milk batches used for cheese production (% *w/v*).

Milk Batches	DM	F	P	L ^1^	A
FFM	11.45 ^a^ ± 0.04	3.35 ^a^ ± 0.01	3.04 ^a^ ± 0.02	4.40 ^a^ ± 0.03	0.66 ^a^ ± 0.00
RFM	9.91 ^b^ ± 0.08	1.41 ^b^ ± 0.03	3.18 ^b^ ± 0.02	4.63 ^b^ ± 0.03	0.69 ^b^ ± 0.00
RFM + CB	10.03 ^b^ ± 0.04	1.43 ^b^ ± 0.02	3.22 ^b^ ± 0.01	4.68 ^b^ ± 0.02	0.70 ^b^ ± 0.00
RFM + CW	9.63 ^c^ ± 0.16	1.17 ^c^ ± 0.03	3.17 ^b^ ± 0.05	4.61 ^b^ ± 0.07	0.69 ^b^ ± 0.01
RFM + CS	9.84 ^bc^ ± 0.14	1.22 ^bc^ ± 0.05	3.23 ^b^ ± 0.03	4.69 ^b^ ± 0.05	0.70 ^b^ ± 0.01

(FFM): full-fat milk; (RFM): reduced-fat milk; (RFM + CB): reduced fat milk plus UF concentrated buttermilk; (RFM + CW): reduced fat milk pus UF concentrated whey; (RFM + CS): reduced fat milk plus UF concentrated sheep’s second cheese whey; (DM): dry matter; (F): fat; (P): protein; (L): lactose; (A): ash. Means within the same column with different superscripts are significantly different (*p* < 0.05) (^1^ calculated by difference).

**Table 3:** Sensory evaluation of the cheese samples at the 30th, 60th and 90th days of ripening.

Cheese	AP 30	AR 30	TE 30	TA 30
FF	7.00 ^a^ ± 1.17	6.63 ^ab^ ± 1.85	7.03 ^a^ ± 1.22	7.23 ^a^ ± 1.63
RF	7.10 ^a^ ± 1.71	6.63 ^ab^ ± 1.56	6.67 ^ab^ ± 1.73	6.57 ^ab^ ± 1.83
RF + CB	7.50 ^a^ ± 1.17	6.87 ^a^ ± 1.38	7.33 ^a^ ± 1.49	7.27 ^a^ ± 1.26
RF + CW	6.80 ^a^ ± 1.65	6.00 ^ab^ ± 1.60	5.77 ^b^ ± 1.45	5.73 ^b^ ± 1.98
RF + CS	6.63 ^a^ ± 1.50	5.73 ^b^ ± 1.48	5.57 ^b^ ± 1.65	5.57 ^b^ ± 1.94
	**AP 60**	**AR 60**	**TE 60**	**TA 60**
FF	7.53 ^a^ ± 1.20	7.23 ^a^ ± 1.10	7.53 ^ab^ ± 1.20	7.50 ^a^ ± 1.28
RF	7.30 ^a^ ± 0.95	6.70 ^a^ ± 1.42	7.40 ^ab^ ± 1.35	7.27 ^ab^ ± 1.55
RF + CB	7.53 ^a^ ± 0.94	7.03 ^a^ ± 1.19	7.67 ^a^ ± 1.18	7.10 ^ab^ ± 1.27
RF + CW	7.23 ^a^ ± 1.04	6.77 ^a^ ± 1.38	7.00 ^ab^ ± 1.39	6.90 ^ab^ ± 1.32
RF + CS	7.20 ^a^ ± 1.19	6.60 ^a^ ± 1.48	6.63 ^b^ ± 1.73	6.43 ^b^ ± 1.89
	**AP 90**	**AR 90**	**TE 90**	**TA 90**
FF	7.77 ^a^ ± 1.28	7.13 ^a^ ± 1.61	7.37 ^a^ ± 1.69	7.50 ^a^ ± 1.59
RF	7.40 ^ab^ ± 1.04	6.70 ^a^ ± 1.58	6.90 ^ab^ ± 1.54	6.93 ^ab^ ± 1.53
RF + CB	7.43 ^ab^ ± 0.94	6.90 ^a^ ± 1.40	6.97 ^ab^ ± 1.63	7.00 ^ab^ ± 1.51
RF + CW	5.93 ^b^ ± 1.66	6.17 ^a^ ± 2.02	6.00 ^b^ ± 1.82	6.33 ^b^ ± 1.63
RF + CS	6.77 ^ab^ ± 1.36	6.10 ^a^ ± 1.90	6.63 ^ab^ ± 1.52	6.63 ^ab^ ± 1.52

(AP) = appearance; (AR) = aroma; (TE) = texture; (TA) = taste. (FF) full-fat; (RF) reduced-fat; RF + CB: reduced-fat with concentrated buttermilk; RF + CW: reduced-fat with concentrated whey; RF + CS: reduced-fat with concentrated second cheese whey. Means within the same column with different superscripts are significantly different (*p* < 0.05).

**Table 4:** Ranking of the cheese samples at the different periods of ripening. Lower values indicate higher positioning in the ranking.

Cheese	Rank 30	Rank 60	Rank 90
FF	2.23 ^a^ ± 1.30	2.23 ^a^ ±1.22	2.17 ^a^ ± 1.49
RF	2.80 ^a^ ± 1.32	2.97 ^ab^ ± 1.30	2.60 ^ab^ ± 1.35
RF + CB	2.23 ^a^ ±1.07	2.60 ^ab^ ± 1.28	2.90 ^ab^ ± 1.18
RF + CW	3.83 ^b^ ± 1.23	3.50 ^b^ ± 1.46	3.87 ^b^ ± 1.31
RF + CS	3.90 ^b^ ± 1.18	3.70 ^b^ ± 1.37	3.47 ^b^ ± 1.14

(FF): full-fat; (RF): reduced-fat; (RF + CB): reduced-fat with concentrated buttermilk; (RF + CW): reduced-fat with concentrated whey; (RF + CS): reduced-fat with concentrated second cheese whey. Means within the same column with different superscripts are significantly different (*p* < 0.05). (AP) = appearance; (AR) = aroma; (TE) = texture; (TA) = taste. Means within same column with different superscripts are significantly different (*p* < 0.05).
